# Determinants of Mortality in a Neonatal Intensive Care Unit in Athens, Greece: A Case-Control Study

**DOI:** 10.7759/cureus.31438

**Published:** 2022-11-13

**Authors:** Theodoros N Sergentanis, Nikolaos Vlachadis, Eleni Spyridopoulou, Tonia Vassilakou, Eleni Kornarou

**Affiliations:** 1 Department of Public Health Policy, School of Public Health, University of West Attica, Athens, GRC

**Keywords:** neonatal intensive care unit (nicu), low birth-weight, neonates, prematurity, mortality

## Abstract

Introduction: The aim of this study was to investigate associations between death and sociodemographic and clinical determinants in a neonatal ICU (NICU) in Athens, Greece, by means of a case-control study.

Methods: The study was conducted between January 2013 and October 2017 at the NICU of “Panayiotis & Aglaia Kyriakou” Children’s Hospital in Athens, Greece. The NICU subjects that died (case group) during this period (n=49) were compared with a control group of 451 NICU-admitted subjects who survived, during the same period. Potential determinants of mortality were assessed; univariate and multivariate logistic regression analysis was performed; odds ratios (OR) and 95% confidence intervals (95% CIs) were estimated.

Results: Gestational age less than 32 weeks (adjusted OR=4.59, 95%CI: 2.09-10.10), low birth weight (adjusted OR=3.14, 95%CI: 1.43-6.91), emergency transfer (adjusted OR=11.92, 95%CI: 1.57-90.60), cyanosis (adjusted OR=5.20, 95%CI: 2.25-12.01), perinatal asphyxia (adjusted OR=6.96, 95%CI: 3.07-15.75), necrotizing enterocolitis (adjusted OR=3.21, 95%CI: 1.03-9.99), need for oxygen supply, and incubator use emerged as independent predictors of mortality.

Conclusion: Extreme prematurity, low birth weight, necrotizing enterocolitis, and emergency conditions are associated with mortality, despite progress made in the field of neonatal intensive care.

## Introduction

Infant mortality is a major public health issue worldwide. The infant mortality rate, defined as the annual number of infant deaths in a country's population per 1000 live births, is a metric that reflects the overall quality of health services and the socioeconomic level of the population in a country and is particularly used by public health agents and policymakers. Neonatal mortality is the major contributor to infant mortality, accounting for 70-80% of deaths in the first year of life [1].

Globally, about 4 million children die each year before the completion of the first year of life. In recent decades, a remarkable decline in infant mortality has been achieved, from 65 deaths per 1,000 live births in 1990 to 29 infant deaths per 1,000 live births in 2018 [2]; however, there are large variations among different countries and social inequalities in infant mortality have persisted [3-5].

In developed countries, the main risk factors affecting infant mortality are preterm birth, low birth weight, and congenital malformations, whereas infections, birth asphyxia, injuries, maternal pregnancy complications, and sudden infant death syndrome account for the remainder of infant deaths [6].

The infant mortality rate in Greece is at low levels; however, the declining trends in the first decade of the 21st century have stagnated over the last decade due to the negative impact of the recent economic crisis in the country [7]. Moreover, the infant mortality rate in Greece seems to be closely linked to the high rates of premature births and advanced maternal age in the country [8-11].

In a small number of published studies, some researchers have attempted to present the recent trends of infant mortality in the Greek population; however, there is a gap in the literature regarding the epidemiological profile of neonatal and infant mortality in the country [7,12]. The aim of the present case-control study in a large neonatal ICU (NICU) in Athens, Greece, was to evaluate associations with various sociodemographic and clinical determinants.

## Materials and methods

This study was conducted in the NICU of the “Panayiotis & Aglaia Kyriakou” Children’s Hospital in Athens, Greece, which is one of the two pediatric hospitals in the city. The study has been approved by the local Institutional Ethics Committee. A case-control design was implemented among the infants hospitalized during the January 2013 - October 2017 period in the hospital. The case group consisted of all infants who died (n=49), whereas the control group consisted of 451 infants who were hospitalized in the NICU and survived. All data were retrospectively reviewed.

The following parameters were abstracted from the hospital archives: gender, gestational age at birth (term births: after 37 completed gestational weeks, early preterm births: at 34-36 weeks gestation, moderate preterm births: at 32-33 weeks, and extreme/very preterm births: before the completion of 32 gestational weeks), birth weight (low birth weight: <2,500 g, normal birth weight: 2,500-3,999 g, macrosomia: ≥4,000 g), multiple pregnancy, birthplace (Athens or outside Athens), hospital of birth (private or public), method of transfer to the Unit (by the parents or by emergency), cyanosis, gestational diabetes, gestational hypertension, maternal substance use, maternal hepatitis B (HBV) status, chorioamnionitis, neonatal jaundice, perinatal asphyxia, respiratory distress syndrome, necrotizing enterocolitis, and use of oxygen supply and use of incubator during hospitalization.

Categorical variables were expressed as absolute and relative frequencies and were compared using chi-squared or Fisher’s exact test, as appropriate. Univariate and multivariate logistic regression analysis was performed. The case-control status was set as the dependent variable; the determinants were set as the independent variables and odds ratios (OR) with 95% confidence intervals (95% CI) were estimated. A core model including extreme/very preterm birth (<32 vs. ≥ 32 weeks gestational age) and low birth weight (<2500 vs. ≥ 2500 g) was the basis of the multivariate analysis. At the multivariate analysis, the effects of determinants proven significant at the univariate analysis were adjusted for the aforementioned two variables (extreme/very preterm birth and low birth weight) of the core model. The level of statistical significance was set at 0.05. Statistical analysis was performed with Stata/SE version 16.1 software (Stata Corp., College Station, Texas, USA).

## Results

Descriptive statistics of the case-control study are shown in Table 1.

**Table 1:** Associations of potential determinants with death in the neonatal ICU (NICU).

Variables	Death				P-value
	No (n=451, control group)		Yes (n=49, case group)		
	n	%	n	%	
Sex					0.282
Male	268	59.4	33	67.4	
Female	183	40.6	16	32.6	
Gestational age (weeks)					<0.001
<32	35	7.8	22	44.9	
32-33	32	7.1	5	10.2	
34-36	97	21.5	4	8.2	
≥37	287	63.6	18	36.7	
Birth weight (g)					<0.001
<2500	129	28.6	35	71.4	
2500-3,999	307	68.1	14	28.6	
≥4000	15	3.3	0	0.0	
Multiple pregnancy					0.249
No	417	92.5	43	87.8	
Yes	34	7.5	6	12.2	
Birthplace					0.316
Outside Athens	122	27.0	10	20.4	
Athens	329	73.0	39	79.6	
Hospital of birth					0.092
Public	327	72.5	41	83.7	
Private	124	27.5	8	16.3	
Transfer to unit					<0.001
By parents	152	33.7	1	2.0	
By emergency	299	66.3	48	98.0	
Cyanosis					<0.001
No	425	94.2	35	71.4	
Yes	26	5.8	14	28.6	
Gestational diabetes					0.496
No	428	94.9	48	98.0	
Yes	23	5.1	1	2.0	
Gestational hypertension					>0.999
No	446	98.9	49	100.0	
Yes	5	1.1	0	0.0	
Maternal substance use					0.463
No	446	98.9	48	98.0	
Yes	5	1.1	1	2.0	
Maternal hepatitis B (HBV)					0.433
No	434	96.2	46	93.9	
Yes	17	3.8	3	6.1	
Chorioamnionitis					0.007
No	446	98.9	45	91.8	
Yes	5	1.1	4	8.2	
Neonatal jaundice					0.295
No	230	51.0	29	59.2	
Yes	221	49.0	20	40.8	
Perinatal asphyxia					<0.001
No	426	94.5	33	67.4	
Yes	25	5.5	16	32.6	
Respiratory distress syndrome					<0.001
No	374	82.9	27	55.1	
Yes	77	17.1	22	44.9	
Necrotizing enterocolitis					<0.001
No	442	98.0	41	83.7	
Yes	9	2.0	8	16.3	
Oxygen supply					<0.001
No	305	67.6	6	12.2	
Yes	146	32.4	43	87.8	
Incubator use					<0.001
No	156	34.6	2	4.1	
Yes	295	65.4	47	95.9	

The majority of subjects in the case group were born before 32 weeks (n=22, 44.9%, extreme/very preterm birth), whereas the majority of subjects in the control group (n=287, 63.6%) pertained to full-term births (≥37 weeks); gestational age was associated with infant death (p<0.001). Low birth weight was more frequent in the case group (71.4% vs. 28.6%, respectively; p<0.001). Emergency transfer to the Unit (98.0% vs. 66.3%; p<0.001), cyanosis (28.6% vs. 5.8%; p<0.001), chorioamnionitis (8.2% vs. 1.1%; p=0.007), perinatal asphyxia (32.6% vs. 5.5%; p<0.001), respiratory distress syndrome (44.9% vs. 17.1%; p<0.001), necrotizing enterocolitis (16.3% vs. 2.0%; p<0.001), oxygen supply (87.8% vs. 32.4%; p<0.001) and incubator use (95.9% vs. 65.4%; p<0.001) were associated with death status. On the other hand, gender (p=0.282), multiple pregnancy (p=0.249), birthplace (p=0.316), public or private hospital of birth (p=0.092), gestational diabetes (p=0.496), gestational hypertension (p>0.999), maternal substance use (p=0.463), maternal HBV status (p=0.433), neonatal jaundice (p=0.295) were not associated with death status.

The results of univariate and multivariate logistic regression analysis are shown in Table 2.

**Table 2:** Results of univariate and multivariate logistic regression analysis examining associations of infant death with determinants; those proven significant in the univariate analysis are shown.

Variable	Compared categories	Unadjusted OR (95% CI)	p-value	Adjusted OR (95% CI)	p-value
Core model					
Extreme or very preterm birth	<32 vs. ≥ 32 weeks gestational age	9.68 (5.00-18.74)	<0.001	4.59 (2.09-10.10)	<0.001
Low birth weight	<2500 vs. ≥ 2500 g	6.17 (3.21-11.85)	<0.001	3.14 (1.43-6.91)	0.004
Additional variables					
Transfer to unit	By emergencies vs. by parents	24.40 (3.34-178.49)	<0.001	11.92 (1.57-90.60)	0.017
Cyanosis	Yes vs. no	6.54 (3.13-13.64)	<0.001	5.20 (2.25-12.01)	<0.001
Chorioamnionitis	Yes vs. no	7.93 (2.06-30.59)	0.003	1.66 (0.38-7.24)	0.503
Perinatal asphyxia	Yes vs. no	8.26 (4.02-16.98)	<0.001	6.96 (3.07-15.75)	<0.001
Respiratory distress syndrome	Yes vs. no	3.96 (2.14-7.31)	<0.001	0.86 (0.36-2.08)	0.740
Necrotizing enterocolitis	Yes vs. no	9.58 (3.51-26.17)	<0.001	3.21 (1.03-9.99)	0.044
Oxygen supply	Yes vs. no	14.97 (6.23-35.97)	<0.001	8.46 (3.31-21.66)	<0.001
Incubator use	Yes vs. no	12.42 (2.98-51.84)	<0.001	6.18 (1.41-27.04)	0.016

At the core model encompassing mutual adjustment between extreme/very preterm birth and low birth weight status, both variables were significantly associated with infant death (adjusted OR=4.59, 95% CI: 2.09-10.10 for extreme/very preterm birth; adjusted OR=3.14, 95% CI: 1.43-6.91 for low birth weight.

After adjustment for extreme/very preterm birth and low birth weight, emergency transfer to the unit (adjusted OR=11.92; 95% CI: 1.57-90.60), cyanosis (adjusted OR=5.20; 95% CI: 2.25-12.01), perinatal asphyxia (adjusted OR=6.96; 95% CI: 3.07-15.75), necrotizing enterocolitis (adjusted OR=3.21; 95% CI: 1.03-9.99), need for oxygen supply (adjusted OR=8.46; 95%CI: 3.31-21.66) and incubator use (adjusted OR=6.18; 95% CI: 1.41-27.04) were positively associated with infant death.

On the other hand, chorioamnionitis (adjusted OR= 1.66; 95% CI: 0.38-7.24) and respiratory distress syndrome (adjusted OR= 0.86; 95% CI: 0.36-2.08) lost their significance in the multivariate analysis adjusting for prematurity and low birth weight.

## Discussion

This case-control study highlighted the independent associations of predictors of death in the context of a NICU. Extreme/very preterm birth, low birth weight, emergency transfer to the unit, cyanosis, perinatal asphyxia and necrotizing enterocolitis, need for oxygen supply, and incubator use emerged as independent predictors of infant mortality, with sizeable effect estimates.

Low birth weight and prematurity are well-established risk factors of severity in the NICU setting; neonatal disease severity scores have included those factors in the core variable set. A systematic review published in 2017 summarizing model parameters of prognostic models for predicting in-hospital neonatal mortality recorded the presence of gestational age and birth weight in SNAP (Score for Neonatal Acute Physiology) models [13]. Afterward, the modified sick neonatal score (MSNS), introduced in 2019, included gestational age (<32 weeks; 32-36 weeks) and low birth weight (<1.5 kg; 1.5-2.49 kg) in its score system encompassing eight parameters [14].

This case-control study showed independent associations between neonatal death and necrotizing enterocolitis. The latter has been acknowledged as a major cause of morbidity and mortality in the NICU; a recent systematic review and meta-analysis showed an incidence of the condition equal to 7% of all very low birth-weight infants [15]. Preterm birth and low birth weight are risk factors of necrotic enterocolitis, whereas other risk factors include race, congenital pneumonia, asphyxia, meconium aspiration, and rapid advancement of feeding, among numerous documented associations [15-19]. Interestingly, in the present study, necrotizing enterocolitis seemed able to confer additional odds of mortality, after adjustment for its major risk factors, namely prematurity and low birth weight.

Cyanosis, perinatal asphyxia, and the need for oxygen supply reflect the respiratory status of the infant; various respiratory status parameters have been a consistent part of scoring systems for the prediction of neonatal mortality [13]. 

From a health system- and public health policy-related point of view, the emergency transfer to the unit seems of special interest as a predictor of mortality. A qualitative study conducted in neonatologists documented the concerns of the specialists vis-à-vis neonatal transfers, including the need for the readiness of paramedics to undertake the transfers, the need for soundness in the referral processes, and coordination between hospital staff, paramedics, and members of the transport team [20]. The paradigm of Italy, a neighboring country to the present study, highlighted that the implementation and improvement of the Neonatal Emergency Transport (NET) service with centers activated throughout the Italian territory were effective in the reduction of infant mortality, as demonstrated in a study published in 2021 [21]. Such health system infrastructure interventions aiming to reduce infant mortality are in line with the Sustainable Development Goals, whose target is to reduce mortality under five years of age to 25 per 1000 live births globally by 2030 [22].

 Various limitations of this study should be declared. First, associations with potentially meaningful predictors of mortality, such as chorioamnionitis and respiratory distress syndrome did not reach significance; larger samples may well be needed for the documentation of these effects. In addition, the study recruitment period was prior to the COVID-19 pandemic; therefore, any differential effects of the mortality predictors during the COVID-19 era could not be addressed. More elaborate indices, such as small-for-gestation age, or composite severity scores were not available for inclusion in the analysis of the study. Moreover, additional, detailed clinical indices, such as body temperature, pH, blood pressure, oxygen saturation, hypoglycemia, and the presence of seizures, were not available for this study. 

## Conclusions

Preterm birth, low birth weight, emergency transfer to the unit, cyanosis, perinatal asphyxia, necrotizing enterocolitis, need for oxygen supply, and incubator use emerged as independent predictors of mortality in the context of a neonatal intensive care unit. Further longitudinal cohort studies, adjusting for composite scores and addressing any potential added value of the aforementioned set of variables are warranted.
